# Modeling neutral evolution of Alu elements using a branching process

**DOI:** 10.1186/1471-2164-11-S1-S11

**Published:** 2010-02-10

**Authors:** Marek Kimmel, Matthias Mathaes

**Affiliations:** 1 Department of Statistics, Rice University, Houston, TX 77005, USA; 2 Systems Engineering Group, Silesian University of Technology, 44-100 Gliwice, Poland

## Abstract

**Background:**

Alu elements occupy about eleven percent of the human genome and are still growing in copy numbers. Since Alu elements substantially impact the shape of our genome, there is a need for modeling the amplification, mutation and selection forces of these elements.

**Methods:**

Our proposed theoretical neutral model follows a discrete-time branching process described by Griffiths and Pakes. From this model, we derive a limit frequency spectrum of the Alu element distribution, which serves as the theoretical, neutral frequency to which real Alu insertion data can be compared through statistical goodness of fit tests. Departures from the neutral frequency spectrum may indicate selection.

**Results:**

A comparison of the Alu sequence data, obtained by courtesy of Dr. Jerzy Jurka, with our model shows that the distributions of Alu sequences in the AluY family systematically deviate from the expected distribution derived from the branching process.

**Conclusions:**

This observation suggests that Alu sequences do not evolve neutrally and might be under selection.

## Introduction and background

Human genome is a result of 10^9 ^years of evolution. It is very complex and in some respects it is still evolving. This contribution concerns evolution of the so-called Alu elements, which are movable sequences of DNA, very abundant in the human genome. We present a mathematical random process, the Griffiths-Pakes discrete-time branching process with infinite-allele mutations, which is almost ideally suited for modeling of Alu elements proliferation. For the biologically important special case of the linear-fractional offspring distribution we derive semi-explicit expressions for the expected frequency spectra of classes of alleles existing in a given number of copies (an analogue of the Ewens sampling formula). We compare the outcome with Alu-element statistics data.

### Alu repeat sequences

#### Background on Alus

Alu elements belong to the group of transposable or mobile elements, which occupy nearly 45% of the human genome [1]. Within this group of transposable and also highly repetitive elements, LINEs (Long INterspersed Elements) and SINEs (Short INterspersed Elements) form the two largest groups. They occupy 21% and 13% of the human genome respectively [2]. Whereas the LINEs are dominated by L1 elements, the largest and hence most studied group of the SINEs is comprised of the Alu elements. While many transposable elements are present in all eukaryotic genomes, Alu elements appear only in mammals. A typical full-length Alu sequence is approximately 300 bp long. Alu sequences amplify by retrotransposition, also known as "the copy and paste" mechanism. At present it is estimated that more than one million copies of Alu elements occupy about eleven percent of the human genome, and the number of elements seems to be growing [1].

Alu elements are non-autonomous and seem to have to use the L1 elements' tools for retrotransposition. It has been hypothesized that L1 endonuclease causes a nick at the TTAAAA consensus site, after which Alu anneals directly to the site of integration [3]; then a second nick on the other strand completes the insertion. These two staggered nicks introduce an identifiable characteristic of Alu elements. The newly inserted Alu element is surrounded by an identical set of direct repeats, which are also called target site duplications (TSDs). These direct repeats range from 10 to 15 bp and are considered the prevalent feature of retrotranspositional insertion [4]. This process of integration, also known as target-primed reverse transcription (TPRT) [5,6], is responsible for the successful amplification of Alu elements. At present it is estimated that more than one million copies of Alu elements occupy about eleven percent of the human genome, and the number of elements seems to be growing [1].

Based on diagnostic mutations, Alu elements are divided into subfamilies. The three major families of Alu sequences are J, S and Y. The letters are chosen in alphabetical order to convey the different ages of each family. Alu sequences in the J family are the oldest, while Alu sequences in the Y family are the youngest. The most interesting family in the current research of Alu elements is the Y family, which contains the youngest and most active Alu elements [7]. Due to their recent integration, 25 percent of their loci are still polymorphic [1]. An Alu locus is defined to be polymorphic if some individuals have an Alu element at that particular location while others do not. These polymorphic loci can be used as genetic markers for disease association studies.

Unlike Single-Nucleotide Polymorphisms (SNPs) Alu markers are small in numbers, but they are identical by descent and essentially homoplasy-free markers and their ancestral state, which is defined by their absence from a specific locus, is always known. Polymorphic Alu loci have been used in genetic diversity studies, forensic studies and disease association studies [8,9]. Alu insertions have influenced the architecture of human genome by duplication, deletion, inversion, transduction and translocation [10]. Alu elements frequently appear in introns, 3' untranslated regions of genes, and intergenic genomic regions [11]. Alu insertions act as insertional mutagens and are responsible for 0.5 percent of human genetic disorders [12]. Almost all these diseases are caused by Alu elements from the youngest subfamilies [6]. For a comprehensive list of AluY disease loci and their associated diseases, one can also consult [6]. Their summary of Alu insertion induced diseases includes neurofibromatosis, hemophilia A and B, Huntington disease and Apert syndrome. Deiniger and Batzer [12] attribute diseases such as insulin-resistant diabetes type II, Lesch-Nyhan syndrome, Tay-Sachs disease, familial hypercholesterolaemia and -thalassaemia to Alu-mediated recombination. Additionally, several types of cancer, including Ewing sarcoma, breast cancer and leukemia are shown to be caused by Alu elements [1,12].

#### Alu sequence data used in this study

Dr. Jerzy Jurka of the Genetic Information Research Institute (GIRI) kindly provided Alu sequence data for our analysis. All Alu subfamilies were extracted from the March 2006 assembly of the USCS Human Genome database. Only recognizable full-length Alu sequences were retained for analysis. Overall, Alu sequences for nine different Alu subfamilies were extracted from the USCS reference genome: AluYa1, AluYa4, AluYa5, AluYa8, AluYb8, AluYc1, AluYd2, AluYe2, and AluYe5.

The goal was to extract Alu sequences that belonged to relatively large subfamilies (more than 1000 sequences), such as AluYa1, AluYa4, AluYb8, AluYc1, and AluYe2. For each subfamily, a consensus or reference Alu sequence was used to screen the entire human genome for matching sequences. A match occurred when stretches of nucleotides that include the main diagnostic mutations agreed with the Alu subfamily consensus sequences. Since the insertion mechanism of an Alu element introduces large differences in their poly-A tails, these need to be deleted from analysis. Dr. Jurka provided the Alu sequence data with poly-A tails already deleted.

Alu sequences contain the middle A-stretch, another highly variable region similar to the poly-A tail, which lies between the two monomers that constitute an Alu sequence, and can be considered the A-tail of the first monomer. To accurately delete the middle-A stretch, it is necessary to align the Alu sequences for each subfamily. A consensus sequence for each subfamily was obtained from Repbase [13], a database of repetitive elements, which is maintained by GIRI. In each subfamily, pairwise alignment of each Alu sequence in the subfamily with the Repbase consensus sequences, was performed using ClustalW [14]. MEGA4 software [15] was used to display the alignments including the middle-A stretch. After deleting the middle-A stretch, the average length of an Alu sequence is about 260 base pairs.

Following preparatory steps described above, we obtained the counts of Alu sequences that had *n *identical copies in the sample, for *n *= 1, 2, 3,.... To obtain these counts for each Alu subfamily, a program was written in R-language. These counts or corresponding percentages represent final data, which were tested against the theoretical distribution based on the branching process model.

## Results and discussion

### Maximum-likelihood fits

To fit the branching process model to the Alu sequence data, we use the maximum likelihood method. The highest value of the likelihood determines the estimates for our parameters. Since the log-likelihood of the sample does not exist in a closed form, we evaluate it numerically. For these runs we set the value of the probability of mutation at *μ *= 10^-6^. Sensitivity of the outcome to variation in parameter *μ *is very slight as long as this parameter is small (such as 10^-5 ^- 10^-9 ^per division).

**Figure 1:**
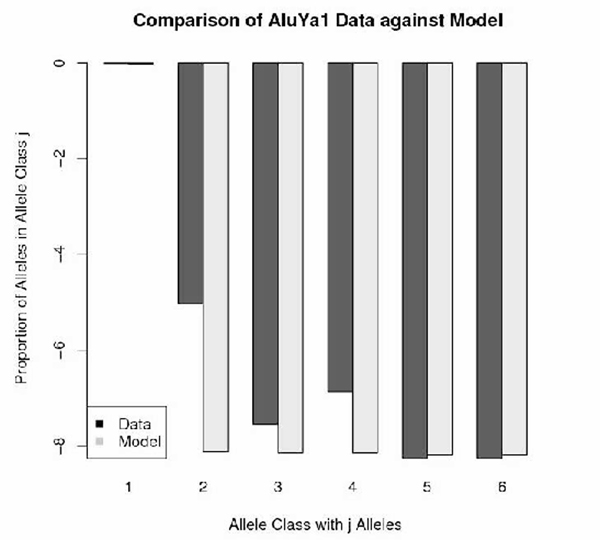
**AluYa1 data-based class frequencies against the theoretical {Ψ_*k*_} in log scale**. Fitted by Griffiths-Pakes process with linear-fractional distribution, with *b *= 0.016, *p *= 0.983.

**Figure 2:**
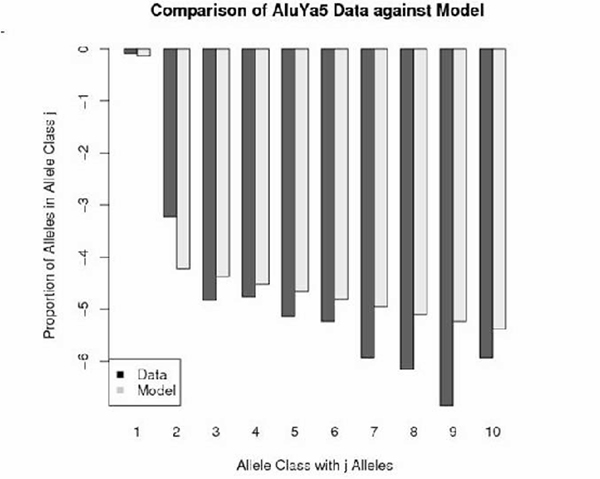
**AluYa5 data-based class frequencies against the theoretical {Ψ_*k*_} in log scale**. Fitted by Griffiths-Pakes process with linear-fractional distribution, with *b *= 0.139, *p *= 0.861.

**Figure 3:**
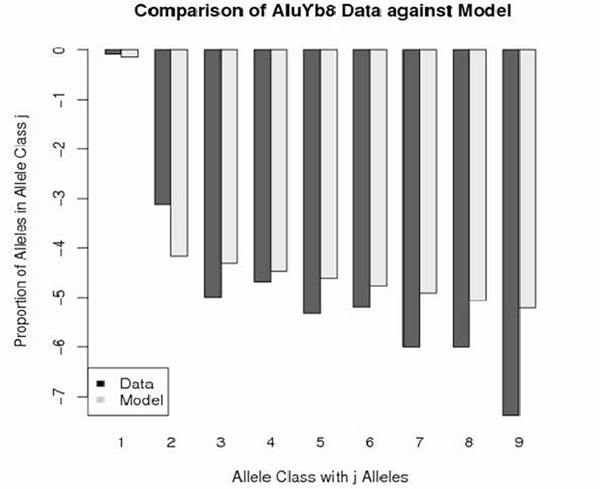
**AluYb8 data-based class frequencies against the theoretical {Ψ_*k*_} in log scale**. Fitted by Griffiths-Pakes process with linear-fractional distribution, with *b *= 0.143, *p *= 0.856.

**Figure 4:**
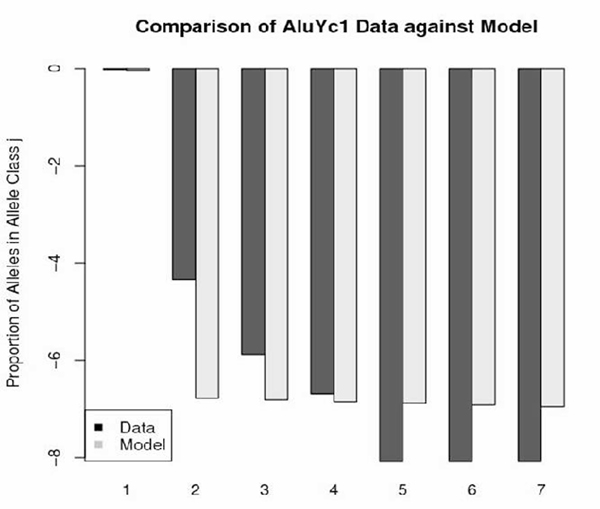
**AluYc1 data-based class frequencies against the theoretical {Ψ_*k*_} in log scale**. Fitted by Griffiths-Pakes process with linear-fractional distribution, with *b *= 0.035, *p *= 0.965.

Figures 1, 2, 3, 4 depict the maximum-likelihood fits of the model to the data from AluYa1, AluYa5, AluYb8 and AluYc1 subfamilies, respectively. They are presented in the semi-logarithmic scale, to amplify the tail probabilities. The graphical comparison demonstrates that the data fit relatively well for allele classes 1 and 3 - 7. Notably, the allele class 2 shows the worst fit among the first seven allele classes. These seven classes account for at least 0.99 cumulative class frequency observed in the data.

### Simulation-based test

Testing for significance of the differences between the theoretical and observed frequencies of allele classes is in our case made difficult by the dominance of class 1 frequency. This causes that, with finite sample sizes, counts of alleles in the further classes are quite low (see Table 1). Therefore the usual restrictions for minimum number of observations and minimum number of classes in the *χ*^2 ^test (as well as in other usual tests for distribution comparisons) are met only in two data sets. For this reason, we resort to a simulation-based approach.

**Table 1:** Frequencies of alleles (mutant types) with *j *copies (class *j *alleles). Classes with *j *> 20 have been omitted.

Number of copies	1	2	3	4	5	6	7	8	9	10	11	12	13	14	16	18
Ya1	3761	25	2	4	1	1										
Ya4	426	6	2	2	1									1		
Ya5	1722	75	15	16	11	10	5	4	2	5	1	1	2	1		2
Ya8	28	3							1							
Yb8	1489	71	11	15	8	9	4	4	1		1		1	1	1	
Yc1	3162	42	9	4	1	1	1		1							
Yd2	401	1														
Ye2	1130	3	1													
Ye5	853	10	7	2	1											
All	12970	237	47	43	23	21	10	8	5	5	2	1	3	3	1	2

In this approach we use the *χ*^2 ^statistic as our test statistic. We start with randomly drawing a sample of size *n *from the uniform distribution *U*(0, 1), where *n *is equal to the number of Alu sequences in each subfamily. Based on the distribution {Ψ_*j*_} produced by our fitted model we obtain the probabilities of Alu sequences with *j *= 1, 2, 3,... copies. These probabilities determine the bin a random draw from the uniform distribution will be placed in. Repeating this *n *times results in a distribution of counts per bin. From this simulated distribution of counts we compute the *χ*^2 ^statistic by using the expected counts under our fitted model and the counts from the simulated (observed) approach. For each Alu subfamily, this process was repeated 100, 000 times. The *χ*^2 ^values were sorted and then plotted to display their distribution. When comparing the *χ*^2^statistic from the actual data to the simulated *χ*^2 ^statistics for each subfamily (Table 2), it becomes apparent that the data produce a very high *χ*^2 ^value, which is highly unlikely under the proposed model. The two Alu subfamilies that have *χ*^2 ^values less than the maximum simulated *χ*^2 ^values are AluYa8 and AluYd2. A closer look, however, reveals that both of the *χ*^2 ^values for the AluYa8 and AluYd2 subfamilies are among the ten highest values in the sample of 100, 000 simulated *χ*^2 ^values (crude *p*-value of 10^-4^). The large *χ*^2 ^statistic for our data is mostly due to the difference between the observed and the expected counts in bin number 2 (Alu sequences with 2 copies) and in the combined bins of the tail. We notice it as a systematic departure.

**Table 2:** Sample-based vs. simulation-based *χ*^2 ^statistic for the Alu subfamilies considered

Alu	Sample-based *χ*^2 ^statistic	Maximum of the simulation-based *χ*^2 ^statistics
Ya1	1118.072	22.72153
Ya4	99.01778	19.06578
Ya5	338.4337	12.60890
Ya8	15.82118	23.83412
Yb8	274.0389	14.98851
Yc1	908.3557	15.46991
Yd2	224.6298	225.5328
Ye2	421.1556	89.49312
Ye5	169.5426	20.00121

For each subfamily 100, 000 simulations were performed.

## Conclusion

The current study seems to constitute the first application of the Griffiths-Pakes process to biological data. The outcome is interesting in the sense that a generally plausible fit is obtained to the Alu element frequency distribution. It is not quite clear, why the fit fails worst at the frequency class 2. This may be influenced by initial steps of data preparation. If a region containing a a relatively frequent variant were removed so that sequences could be aligned, some unique variants might migrate to class 2. Another possibility is that the difference is caused by a departure from neutrality in Alu evolution.

We should notice that the current model does not involve genetic drift. In reality, the genomes evolve within individuals and properly, the branching process should have been embedded in a population genetic model of Wright-Fisher or Moran type. However, this would lead to enormous complications. As an additional exercise, we attempted to fit the Alu class frequencies by the classical Ewens sampling formula, using a test developed by Slatkin [16], but the fit is rather bad.

## Methods

### Discrete branching process of Griffiths and Pakes with infinite allele mutations

Branching processes have been widely used in modeling cell population dynamics. An insertion of an Alu sequence into a new genomic location can be considered a proliferation process not dissimilar from cell division. Therefore proliferation and mutation of Alu sequences can be described in a mathematical way using a branching process. The branching process has to account for the fact that Alu sequences are still growing in numbers in the human genome. Therefore we focus on the supercritical branching processes, in which the expected number of offspring is greater than one (*m *> 1). One interesting model prediction, which can be compared to data is how many different Alu sequences occur in each Alu subfamily or more specifically how many Alu alleles with frequency *j *exist in each subfamily. Based on a discrete-time branching process with infinite allele mutations, Griffiths and Pakes [17] derived a limit result for the expected proportion of alleles having frequencies in *j*.

**Figure 5:**
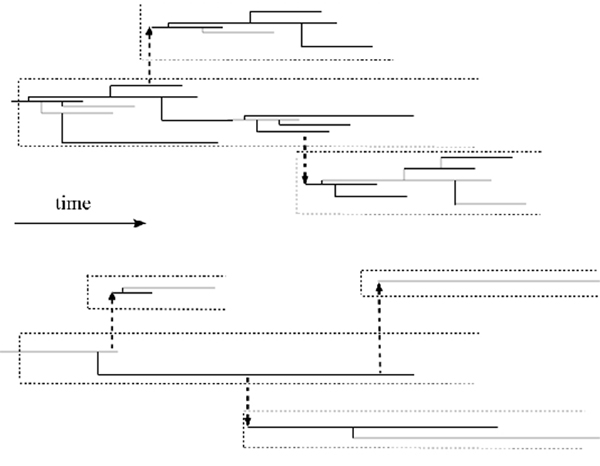
**Griffiths-Pakes branching process with infinite-allele mutations**. A non-mutant clone of particles is evolving in time according to a single-type branching process (in our case, time dicrete). With probability *μ *per time step, a particle mutates and initates a clone of new previously nonexistent type, which evolves according to the same rules as the original non-mutant clone. As a result, a set of clones of different types emerges, spawning further clones, some of which may die out. Upper panel: low *μ*; lower panel: high *μ*.

Griffiths and Pakes [17] process is a modification of the standard Bienayme-Galton-Watson branching process to allow individuals infinitely many possible identifiable types. In our application, the types are alleles (variants) of the Alu sequence identified by specific point mutations. From time *t *= 0, a non-mutant clone of particles is evolving in time according to a single-type branching process (Figure 5). With probability *μ *per time step, a particle mutates and initates a clone of new previously nonexistent type, which evolves according to the same rules as the original non-mutant clone. As a result, a set of clones of different types emerges, spawning further clones, some of which may die out. We are interested in deriving, using Griffiths-Pakes [17] theory, expected frequencies of allele classes such that allele is in class *k *if it exists in *k *copies, for a specific biologically justifiable version of the process.

The number of individuals at *t *= 0 is defined as *Z*_0 _= *i*. Let *G*_*n *_be the collection of individuals in generation *n *and let *Z*_*n *_denote their number. Each generation size depends on the previous generation size through the branching property

where *ξ*_*j*,*n *_are independent identically distributed (iid) integer-valued random variables, which represent the number of offspring born to the *j*^*th *^member of *G*_*n*_. The distribution of *ξ*_*j*,*n *_is characterized by its probability generating function (pgf)

where *p*_*k *_= *P*[*ξ*_*j*,*n *_= *k*], and it is assumed that *p*_0 _+ *p*_1 _*<*1, i.e., the branching process is nontrivial. We have *m *= *f'*(1).

If an individual produces *j *offspring then the number of progeny having the parental allele is distributed binomially with parameters *j *and 1 - *μ*, hence its pgf is equal to (*μ *+ (1 - *μ*)*s*)^*j*^. This implies that any new allele is followed by a branching process of its like-type descendants with offspring pgf *H*(*s*) = *f*(*μ *+ (1 - *μ*)*s*). This process is supercritical if its expected progeny count *M *= *m*(1 - *μ*) is greater than 1. Within this framework let us define the symbol , where *H*^(*r*)^(*s*) is the *r*th iterate of pgf *H*(*s*), to be equal to the probability that there are *j *individuals at time *r *in a nonmutant clone started at time 0 by a single individual. Let us denote Ψ_*j *_the long-term expected proportion of alleles with frequency *j *≥ 1, which is the formula that we will use to compute the theoretical distribution of Alu allele classes for given offspring pgfs. Asymptotically, these proportions assume the form (based on Griffiths and Pakes [17], detailed derivation in [18]

### Linear fractional offspring distribution

The process of creation of new Alu repeats by retrotransposition can be naturally described by the age dependent Markov branching process {*Z*_*t*_} (i.e., process with exponentially distributed individuals' lifelengths) with binary fission, which leads to a quadratic pgf of progeny number per individual. The rationale is that any existing Alu ("individual") from an active family produces two progeny (i.e., itself and a replica) at a random time time moment, where "random" means that the intervals between successive fission events are independent, identically distributed random variables. Moreover, the copy may fail to reinsert into the genome. Therefore, the form of the progeny count pgf will be *αs*^2 ^+ (1 - *α*)*s*, where *α *is the probability of successful reinsertion. If such process is sampled at constant time intervals, the resulting discrete-time process {*Z*_*k*Δ*t*_} is a Galton-Watson branching process with linear fractional pgf ([19], expression (4.14), also c.f. [20]). A unique property of the linear fractional case of the Galton-Watson process, excluding the trivial case *f*(*s*) = *ps *+ *q*, is that the iterations of the pgf can be computed explicitly and also are of linear fractional form. Let us start with the offspring pgf in the linear fractional case:

The probability distribution corresponding to this generating function is:

The parameters *b *and *p *are subject to certain restrictions,

To ensure that this process is supercritical, i.e., *m *> 1, additional constraints on *b *and *p *are needed. The mean of *f*(*s*) is , so supercriticality yields an additional restriction on parameters *b *and *p*, *b *> (1- *p*)^2^, or equivalently

To be more precise, we should satisfy condition *m*(1 - *μ*) > 1, but with *μ *very close to 0, the distinction is not important. As demonstrated in [18], for the linear-fractional case, we obtain the following computable expression

**Figure 6:**
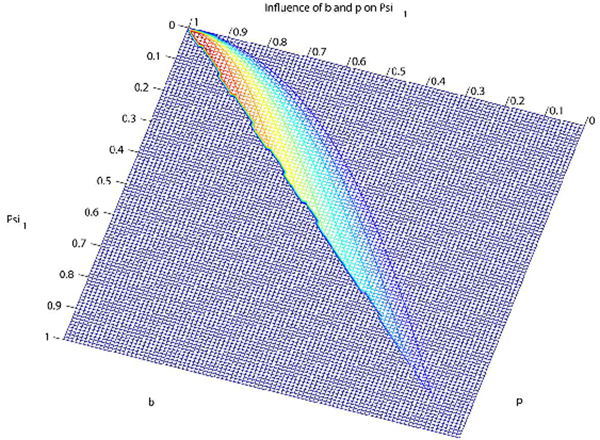
**Contour plot illustrating the influence of parameters *b *and *p *on Ψ_1_, based on Griffiths-Pakes process with linear-fractional distribution**. Red: large Ψ_1_; blue: small Ψ_1_. Range of Ψ_1_-values, from 0 through 1.

The infinite sums in the numerator and denominator are numerically computed. A program was written in R-language to compute the Ψ_*j*_. Since Alu sequence data in Table 1 suggest a high value for Ψ_1_, we verify that the theoretical Ψ_1 _attains such values for any choices of parameters *b*, *p*, and *μ*. For fixed *μ *= 10^-6^, we established a grid of *b *and *p *from 0 to 1 in steps of 0.01. Figure 6 shows that Ψ_1 _can assume any value between 0 and 1, and that high values of Ψ_1 _occur for a combination of low values of *b *and high values of *p*.

## Competing interests

The authors declare that they have no competing interests.

## Authors' contributions

MK conceived the study. MK and MM jointly derived the mathematical model equations. MM prepared the Alu element data for model fitting carried out sequence alignment and statistical testing. MK drafted the manuscript with MM's help. All authors read and approved the final manuscript.
